# Development of Microscopy Apparatus Switchable between Fluorescence and Ultralow-Frequency Raman Modes

**DOI:** 10.1155/2022/2694545

**Published:** 2022-09-19

**Authors:** Ken Takazawa

**Affiliations:** National Institute for Materials Science, 3-13 Sakura, Tsukuba 305-0003, Japan

## Abstract

In this study, a microscopy apparatus that can switch between the fluorescence microscopy and ultralow-frequency Raman microscopy modes was developed. The apparatus can be easily constructed by equipping a standard epi-illumination microscope with an additional port featuring a removable half mirror. Owing to the switchability, fluorescence imaging, and spectroscopy, Raman spectroscopy in the frequency range down to ∼10 cm^−1^ can be performed using the apparatus. To demonstrate the advantageous features of this apparatus, micron-sized crystals of perylene, which have two polymorphic forms, were analyzed. The two polymorphs were clearly identified based on their shapes, fluorescence spectra, and ultralow-frequency Raman spectra, all of which can be observed with our apparatus alone. These results indicate that the apparatus is a powerful tool for the analysis and characterization of various nano-/micron-sized crystals.

## 1. Introduction

Ultralow-frequency (ULF) or terahertz Raman spectra in the frequency region below∼100 cm^−1^ have attracted increasing interest for the analysis of various materials, such as organic materials [1], semiconductors [2–5], and biological materials [6, 7]. For instance, ULF Raman spectra provide information on the intermolecular (lattice) vibrations of organic crystals [8, 9] and the shear and breathing vibrations of 2D layered materials [2–5]. This information on the ULF vibrational modes is crucial for understanding the structural and electronic properties of these materials. However, conventional Raman microscopy apparatus consisting of dielectric interference filters, including a laser line filter, dichroic mirror, and long-pass filter, is not capable of measuring the spectra in the ULF region because of the lack of spectral resolution to eliminate the residual laser light in the scattering signal selectively. In the last decade, it has been demonstrated that the use of volume Bragg gratings (VBGs) instead of dielectric interference filters enables the measurement of ULF Raman spectra. VBGs were fabricated in a photo-thermo-reflective glass function as band-pass and notch filters with an ultranarrow spectral bandwidth [10, 11]. Raman apparatus constructed using the VBGs has been reported to achieve measurements in the frequency range down to ∼5 cm^−1^.

However, ULF Raman apparatus using VBGs has not been widely used yet for its applicability in many fields of materials science. This is because the apparatus consisting of VBGs is much complicated than that consisting of dielectric interference filters [12, 13]. Because dielectric interference filters can be mounted in the filter unit of a microscope, a standard microscope can be converted into a Raman apparatus without major modifications. However, VBGs cannot be mounted in such units because they have a large thickness, and they must be aligned at a peculiar angle with respect to the optical axis. Therefore, a specially designed microscopy setup must be constructed.

This study reports that a standard epi-illumination fluorescence microscope equipped with a single monochromator and a charge-coupled-device (CCD) camera can be easily converted to a ULF Raman microscopy apparatus by installing an additional port with a removable half mirror. It is demonstrated that that Raman spectra in the ULF region down to ∼10 cm^−1^ can be measured using this apparatus. Moreover, the modification makes the apparatus switchable between the fluorescence microscopy and ULF Raman microscopy modes simply by inserting and removing the half mirror. Owing to its switchability, the apparatus allows consecutive measurements of the fluorescence images, fluorescence spectra, and ULF Raman spectra of selected individual objects. The advantage of switchability stands out when the apparatus is used for the analysis of nano-/micron-sized crystals because the morphology, electronic properties, and crystal structures of individual crystals can be characterized using the apparatus alone.

To highlight this advantage, the apparatus was used to analyze the microcrystals of perylene, which is a polyaromatic hydrocarbon consisting of four benzene rings. Perylene crystals have two polymorphic forms, *α*- and *β*-crystals. It has been reported that stable *α*-crystals have a square shape, whereas metastable *β*-crystals have a rhombic shape [14–18]. Furthermore, the *α*-crystals emit orange fluorescence upon optical excitation at∼400 nm, whereas the *β*-crystals emit green fluorescence [14–18]. We demonstrate that these two polymorphs can be clearly identified from their shapes, fluorescence spectra, and ULF Raman spectra, and they can all be observed with the apparatus. This result indicates that our apparatus is a powerful tool for the analysis and characterization of various nano-/micron-sized crystals.

## 2. Materials and Methods

### 2.1. Fluorescence Microscopy Mode

**Figure 1:**
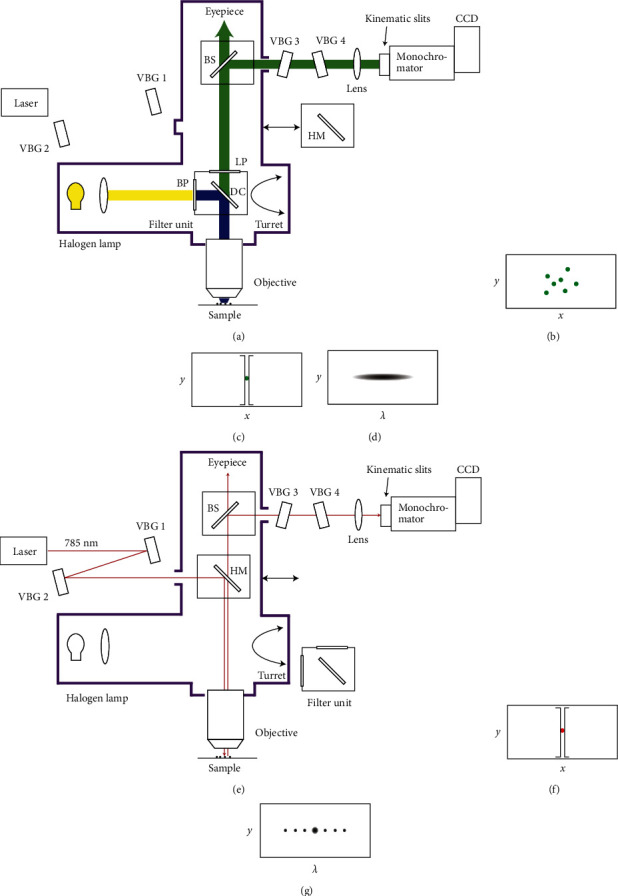
Schematic illustration of the apparatus. The purple line represents the microscope. (a) Fluorescence microscopy mode. VBG: volume Bragg grating. BP: band-pass filter. DC: dichroic filter. LP: long-pass filter. HM: half mirror. (b) Fluorescence image of the sample. (c) Spatial selection of a single crystal by the slits of the monochromator. (d) Fluorescence spectrum of the selected crystal. (e) Ultralow-frequency Raman microscopy mode. (f) Spatial selection of a single crystal by the slits of the monochromator. (g) Raman spectrum of the selected crystal.

Figure 1(a) schematically illustrates the apparatus. The main body of this apparatus was a standard epi-illumination microscope (Olympus, BX-51) equipped with a filter set for fluorescence microscopy, i.e., band-pass, dichroic, and long-pass filters. The filter unit was mounted in a turret and exchangeable with other sets by rotating the turret. An additional port (Olympus, U-DP) equipped with a half mirror (Thorlabs, BSW26 R) was installed above the turret. The half mirror is removable by sliding it out from the optical path. In the fluorescence microscopy mode, the half mirror is removed.

Light from a halogen lamp was introduced into the filter unit and directed to the sample, and the sample was then illuminated through an objective lens (Olympus, 20–100×). The fluorescence from the sample was collected by the same objective lens and passed through the dichroic and long-pass filters, which eliminated the excitation light. A portion of the fluorescence extracted from the microscope by a beam splitter passed through two VBGs (VBG 3 and VBG 4). The transmission properties of the VBGs for fluorescence are discussed later. Thereafter, the fluorescence was imaged onto the slits of a monochromator (Princeton Instruments, SpectraPro 2150, *f* = 150 mm) using an imaging lens (*f* = 200 mm). Two gratings (300 and 1200 g/mm) were installed in the monochromator. The slits of the monochromator are kinematic, and they can be reversibly changed to a 12 mm × 12 mm clear aperture. The fluorescence was detected using a liquid-nitrogen-cooled back-illuminated CCD camera (Princeton Instruments, Spec10, 1340 × 400 pixels) attached to the monochromator. By changing the slits to the open aperture and setting the grating (either 300 or 1200 g/mm) to the zero-order position, the fluorescence image of the sample is recorded in Figure 1(b). When a selected crystal is positioned in the middle of the image area and the clear aperture is changed to slits, the fluorescence from the crystal is spatially selected (Figure 1(c)). The fluorescence spectrum of the crystal was measured by tuning the grating (300 g/mm) to an appropriate angle (Figure 1(d)). The resolution of the fluorescence spectrum was ∼1 nm.

### 2.2. Ultralow-Frequency Raman Microscopy Mode

**Figure 2:**
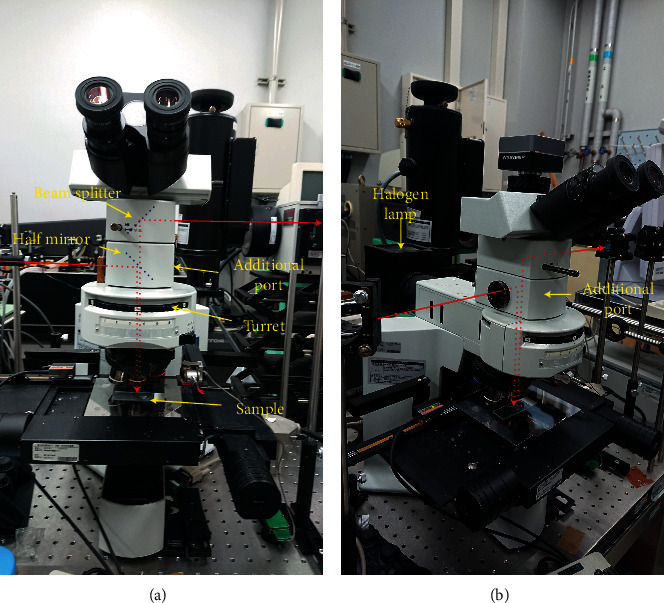
Photographs of the microscope part of the apparatus. Front view (a) and side view (b). The red line represents the path of the laser beam and the scattering. The laser beam introduced into the microscope is directed to the sample by the half mirror mounted in the additional port. The scattering from the sample is passed through the half mirror and extracted from the microscope by the beam splitter.

The filter unit for the fluorescence microscopy was removed from the optical path by rotating the turret, and the half mirror in the additional port was inserted into the optical path (Figure 1(e)). An output of a continuous-wave diode laser at 785 nm (Necsel, SLM-785.0-FS, power: 50 mW) was used for the excitation. The laser beam was consecutively reflected by two VBGs (VBG 1 and 2, OptiGrate, BP-785), which function as ultranarrow laser line filters (bandwidth: ∼5 cm^−1^ (full width at half maximum, FWHM)), and introduced into the microscope through the additional port. Thereafter, the laser beam was directed to the sample by the half mirror and focused to a diffraction-limited spot on the sample by a 100× objective lens (spot size: ∼1 *μ*m). Scattering from the sample was collected by the same objective lens, passed through the half mirror, and extracted from the microscope using a beam splitter. The scattering was then passed through two VBGs (VBG 3 and 4, OptiGrate, BNF-785-OD4-11M), which reflect (block) the residual laser light (bandwidth: ∼5 cm^−1^ (FWHM)), imaged onto the slits by the imaging lens, and detected by the CCD camera through the monochromator. The slit width was 10 *μ*m, and the 1200 g/mm grating was used for the Raman measurement. The obtained image provides spatially resolved Raman spectra of the selected crystal (Figures 1(f) and 1(g)). Photographs of the main body of the apparatus are shown in Figure 2(a) and 2(b).

### 2.3. Key Features of the Apparatus

A key feature that makes our apparatus easy to construct and switchable between the two modes is the use of the half mirror to direct the excitation laser beam to the sample in the ULF Raman mode. In a standard ULF Raman microscopy apparatus, the VBG is used for this purpose. Because the VBG cannot be mounted in the filter unit of a microscope, a specially designed apparatus must be constructed. Therefore, the use of a half mirror significantly contributes to the simplification of our apparatus. However, unlike the VBGs, the half mirror does not selectively reflect the excitation laser. Moreover, it causes a 50% loss of both the excitation laser power and the Raman scattering intensity. However, the ease of construction and switchability of the apparatus outweigh these disadvantages in many applications. One such application is presented in the Results and Discussion section.

The other key future is the use of a laser at 785 nm as the excitation source for the Raman measurement. In our apparatus, VBGs 2 and 3 were placed in the detection path, even in the fluorescence microscopy mode. Because VBGs 2 and 3 reflect (block) light at the wavelength of the excitation laser, the fluorescence at the wavelength is also blocked. To avoid this, a wavelength of 785 nm, which is at the long-wavelength edge of the visible wavelength range, was chosen. Thus, the fluorescence over nearly the entire visible wavelength range does not overlap with the blocking wavelength of the VBGs. However, the transmittance of VBGs is not unity at wavelengths other than the blocking wavelength, and it is wavelength-dependent. Therefore, the fluorescence spectrum measured through the VBGs must be calibrated using their transmittance spectra, as described in the Results and Discussion section.

### 2.4. Sample Preparation

Perylene was purchased from Wako Pure Chemical Industries, and it was used without further purification. Approximately, 2 mM solution of perylene in acetone was drop-cast onto a glass substrate (microscope cover glass, 22 mm × 22 mm), and the solvent was allowed to evaporate under ambient conditions. This procedure resulted in the formation of micron-sized single crystals of perylene.

## 3. Results and Discussion

### 3.1. Transmission Properties of VBGs

**Figure 3:**
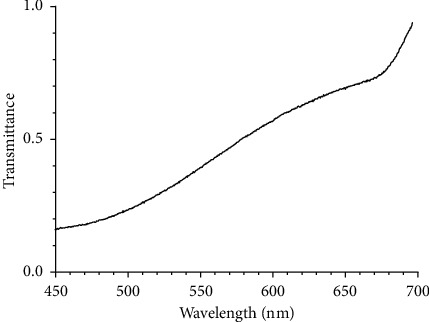
Transmittance spectrum of the two volume Bragg gratings placed in the detection path (VBGs 3 and 4 in Figure 1). Fluorescence spectra measured with our apparatus must be calibrated using this transmittance spectrum.

The transmission properties of the VBGs 3 and 4 were measured using the apparatus in the fluorescence microscopy mode. The half mirror was mounted on the filter unit instead of the filter for fluorescence microscopy. The spectra of light from the halogen lamp reflected by a blank sample were measured with and without the VBGs, and the transmittance spectrum was obtained (Figure 3). The transmittance decreases from 0.92 to 0.18 as the wavelength decreases from 700 to 450 nm. Because there is no rapid change in the transmittance with wavelength, it can be concluded that a valid fluorescence spectrum for this wavelength range can be obtained by calibrating the spectrum using this transmittance spectrum.

### 3.2. Application to Perylene Microcrystals

**Figure 4:**
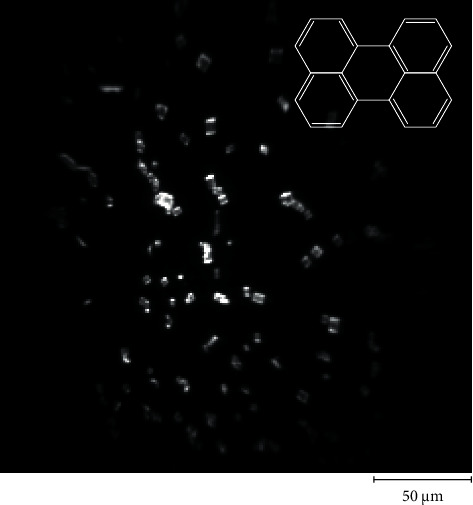
Fluorescence microscope image of the perylene microcrystals on the glass substrate. Micron-sized crystals dispersed on the substrate were observed. Inset: chemical structure of perylene.

**Figure 5:**
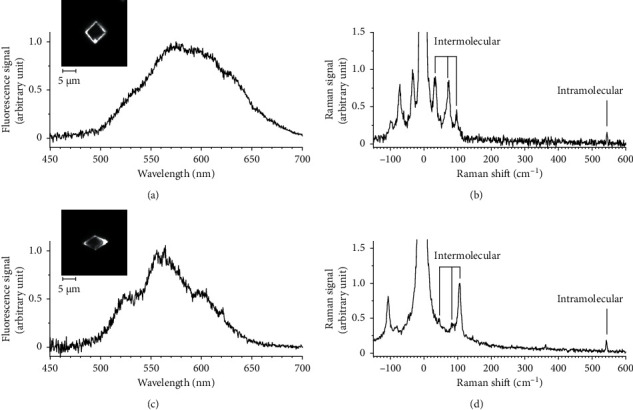
Fluorescence images, fluorescence spectra, and ultralow-frequency Raman spectra of the individual perylene microcrystals. (a) Fluorescence spectrum of a microcrystal with a square shape. Inset: fluorescence image of the crystal. (b) Ultralow-frequency Raman spectrum of the crystal shown in the inset of (b). Three intermolecular vibration modes in the ultralow-frequency region and one intramolecular vibration mode are observed. (c) Fluorescence spectrum of a microcrystal with a rhombic shape. Inset: fluorescence image of the crystal. (d) Ultralow-frequency Raman spectrum of the crystal shown in the inset of (c). Three intermolecular and one intramolecular vibration modes are observed.

First, the sample was investigated via fluorescence microscopy. The apparatus was set in the fluorescence microscopy mode, and a filter set consisting of a band-pass filter (Omega Optical, XF1076, pass band: 385–415 nm), dichroic filter (Omega Optical, XF2040), and long-pass filter (Omega Optical, XF3088, pass band: >435 nm) was equipped.  Figure 4 shows a fluorescence image obtained using a 20× objective lens, showing micron-sized crystals dispersed on the substrate. Most crystals have a square shape, whereas less than one percent of them have a rhombic shape. One of the square-shaped crystals was selected (inset in Figure 5(a), and its fluorescence spectrum was measured. The spectrum calibrated using the transmittance spectrum of the VBGs (Figure 3) shows a broad fluorescence band peaking at *λ*∼580 nm (Figure 5(a)). This spectrum agrees with that reported for *α*-crystals of perylene microcrystals [14–16].

The apparatus was then switched to ULF Raman microscopy mode, and the Raman spectrum of the identical crystal was measured, as in Figure 5(b). The spectrum shows three peaks at 33, 74, and 97 cm^−1^, confirming that the apparatus was capable of ULF Raman measurements. Because VGBs 3 and 4 function as notch filters, both Stokes and anti-Stokes Raman peaks were observed. The spectral resolution was estimated to be ∼6 cm^−1^ from the FWHM of the Raman peaks. These ULF peaks were assigned to intermolecular (lattice) vibration modes [19, 20]. To our knowledge, detailed assignments of these peaks have not been performed yet, and they will be presented in our next paper. In addition to the ULF modes, a peak was observed at 544 cm^−1^. This peak was assigned to the intramolecular C-C stretching vibration mode [20].

Next, a crystal with a rhombic shape was selected (inset in Figure 5(c)), and its fluorescence spectrum was measured. The calibrated spectrum shows a broad band peaking at *λ*∼560 nm, which is blue-shifted with respect to that of the square crystal (*α*-crystals) (Figure 5(c)). This spectrum is consistent with that reported for *β*-crystals [14–17]. Then, the apparatus was switched to the ULF Raman mode, and the Raman spectrum of the identical crystal is measured in Figure 5(d). Three peaks at 44, 87, and 105 cm^−1^ were observed in the ULF region, and the spectral pattern was different from that of the *α*-crystal, indicating that the crystal is a *β*-polymorph. The assignments of these peaks will also be presented in a future paper. The above results show that the two polymorphs of perylene microcrystals were clearly identified from the shapes, fluorescence spectra, and ULF Raman spectra obtained using our apparatus, indicating that it can be a powerful tool for the analysis and characterization of various nano-/micron-sized crystals.

## 4. Conclusions

In this study, a microscopy apparatus that is switchable between the fluorescence and ULF Raman modes was developed. The apparatus was easily constructed by equipping a standard epi-illumination microscope with an additional port with a half mirror. To demonstrate the application of this apparatus, micron-sized perylene crystals, which have two polymorphic forms, were analyzed. Intermolecular (lattice) vibration modes of the perylene microcrystals in the ULF region were observed for the polymorphs. It was demonstrated that the two polymorphs could be identified from the shapes, fluorescence spectra, and ULF Raman spectra, all of which could be observed with the apparatus alone. These results indicate that our apparatus has potential applications in various fields of material science.

## Data Availability

The data that support the findings of this study are available from the author upon reasonable request.
